# The conundrum of discordant protein and mRNA expression. Are plants special?

**DOI:** 10.3389/fpls.2014.00619

**Published:** 2014-11-07

**Authors:** Isabel Cristina Vélez-Bermúdez, Wolfgang Schmidt

**Affiliations:** Integrative Root Development Lab, Institute of Plant and Microbial Biology, Academia SinicaTaipei, Taiwan

**Keywords:** omics, systems biology, post-transcriptional regulation, ribosomes, alternative splicing, gene expression

Rapid progress in transcriptional and proteomic profiling methodologies, such as RNA-sequencing and high-resolution tandem mass spectrometry in combination with nanoflow chromatography, allows for a more accurate comparisons of disparate omics data sets at high resolution. Despite the increased fidelity of such surveys, several studies in mammals, yeast and plants have shown that transcript levels are not always a good proxy for protein abundance. While to some extent the modest concordance observed in studies across platforms may result from fundamentally different protocols that are used for the detection of proteins and mRNAs or caused by differences in the sensitivity of detection and data analysis (“false discordance”), it is becoming obvious that transcript/protein discordance is largely of biological origin (“true discordance”) and represents a critical layer of regulatory processes at the post-transcriptional level that is often neglected.

## The more the better? the concept of potentiation

Gene activity is the result of complex dynamics between the transcription rate of the DNA template, the stability of the mRNA, the translation efficiency of the transcript, and degradation of the protein. Interestingly, the concordance between the abundance of orthologous proteins among related species is higher than that between proteins and their cognate mRNAs within a species (Kwon et al., 2013). This suggests that a certain set point of protein abundance is established to ensure optimal function, although this set point is not necessarily controlled at the transcriptional level. Rather, protein synthesis and feedback inhibition of the synthesis rate appear to dictate protein expression (Kristensen et al., 2013).

Contrary to expectations, direct coupling of transcription and translation, which occurs in prokaryotes, results in lower mRNA/protein concordance when compared to eukaryotes where the two processes are spatially separated (Vogel and Marcotte, 2012). Interestingly, concordance values are highest in single cell eukaryotes and lowest in humans (overview in De Sousa Abreu et al., 2009), indicating that cellular diversity is a large contributor to the difference between mRNA and protein abundance. In particular, decreased mRNA abundance is not conclusive. In a comprehensive survey of changes in protein and transcript profiles in *Arabidopsis* roots in response to phosphate deficiency, we observed a complete lack of correlation between down-regulated transcripts and the amount of their corresponding proteins, whereas for induced genes changes in the levels of mRNAs and proteins were reasonably well correlated (Lan et al., 2012). A similar observation was reported for yeast cells subjected to salt treatment, in which transcript reduction produced only minor changes in the abundance of the corresponding proteins (Lee et al., 2011). Strong induction of transcription is a much better predictor of changes in protein levels than decreased expression. This might be related to the importance of stress-associated proteins that are required to recalibrate cellular metabolism. In fact, highly and lowly transcribed genes differ in their translational fitness, i.e., their association with polysomes (Preiss et al., 2003). Relatively few studies compare mRNA and protein expression in plants using (mainly Arabidopsis and maize), but the general picture that emerges from these studies is a variable correlation of protein–transcript pairs (Baerenfaller et al., 2012; Walley et al., 2013; Ponnala et al., 2014) that is comparable to what has been reported for other organisms. Thus, although technical improvements and the use of techniques measuring *in vivo* translation such as ribosome profiling (Ingolia, 2014) will correct the observed correlations, it appears that at least a substantial proportion of this lack of concordance is of biological origin and thus reflect post-transcriptional regulation rather than technical constrains.

Preferential translation of transcripts derived from highly induced genes, referred to as homo-directional co-regulatory response or potentiation (Preiss et al., 2003), amplifies transcriptional changes at the translational level, leading to a fast and robust change in gene activity. In plants, translation efficiency can change dramatically in response to abiotic stress, leading to a massive bias in the pool of mRNAs that are actively translated (Mustroph et al., 2009; Juntawong et al., 2014). Potentiation of gene expression can be encoded in both DNA and mRNA; translation of *Arabidopsis* mRNAs upon light treatment is dependent on the presence of the sequence motifs TAGGGTTT or AAAACCCT in their 5′ UTR (Liu et al., 2012). These elements are also required for transcription (Tremousaygue et al., 1999; Tatematsu et al., 2005), suggesting that such co-regulatory responses have evolved to switch the expression of genes into a fast-forward mode when the demand for the encoded proteins is high. One may speculate that, while gene induction is amplified by preferred translation of transcripts derived from these genes to secure fast acclimation, down-regulation of gene expression is a weaker signal and the full scale of regulatory post-translational mechanisms results in pronounced discordance between transcripts and proteins.

## Stop making sense: production of non-functional transcripts to tune protein abundance

In principle, the mechanisms that control RNA turnover, translation, and protein stability are similar among eukaryotes. However, lacking behavioral recourses in coping with unfavorable conditions, plants can adjust their developmental, metabolic and physiological programs in a much broader way than, for example, mammals. The phenotypic plasticity of plants results from sophisticated sensing and signaling circuits that integrate disparate environmental cues and modulate gene activity to adjust the phenotypic readout to the prevailing conditions.

The ability of plants to adapt rapidly to a wide range of conditions is reflected by numerous plant-specific peculiarities in the control of gene activity. This high level of transcriptional regulation may correspond to an equally pronounced abundance of post-transcriptional regulatory processes. An interesting example of a process that is seemingly similar in plants and animals, but that has significantly distinct consequences, is the splicing of pre-mRNAs during transcription. In human cells, the vast majority of intron-containing genes (~95%) are alternatively spliced, leading to the generation of multiple, distinct mRNAs from a single gene. The predominant form of alternative splicing in mammals is the exclusion of cassette exons together with its flanking introns, a process referred to as exon skipping, which leaves the open reading frame uninterrupted (Kornblihtt et al., 2013). Exon skipping is thought to contribute to proteome diversity by producing protein isoforms that are structurally and functionally distinct. Other forms of alternative splicing, i.e., introns retention or alternative 5′ and 3′ splice sites, produce transcripts that mostly contain premature stop codons (PTCs), targeting these transcripts for degradation via the nonsense-mediated decay RNA surveillance pathway, a mechanism that prevents the production of truncated proteins by eliminating PTC-containing mRNAs after the pioneer round of translation.

In plants, intron retention and alternative 5′ and 3′ splice sites comprise the majority of alternative splicing events (Filichkin et al., 2010; Marquez et al., 2012; Li et al., 2013; Wu et al., 2014). Splicing patterns appear to be more complex during acclimation to nutrient deficiencies or light exposure than under constant conditions, resulting in significantly induced intron retention features (Li et al., 2013; Wu et al., 2014). The reasons for the different forms and consequences of alternative splicing in animals and plants are currently unknown. Two plausible scenarios would explain the high number of intron- (and, in most cases, PTC-) containing transcripts in plants. Firstly, non-functional mRNAs could be stored as ribonucleoproteins and processed when needed. This would allow for a fast increase in populations of mature mRNAs that code for proteins that are required upon stress exposure or during development. In an alternative, but not mutually exclusive scenario, non-functional transcripts are produced to tune the abundance of proteins with critical functions in response to a fluctuating environment. A quick shift between the production of functional and non-functional transcripts also aids in rapid re-adjustment of protein levels after the stress is relieved. Production of non-functional transcripts would provide an alternative mechanism for such an adjustment, circumventing changes in transcription rates, which involve recruitment/dismissal of transcription factors and changes in chromatin structure.

Changes in splicing patterns in plants as a means to calibrate protein abundance would imply a feedback mechanism that communicates the demand for a given protein to the splicing machinery, and a switch to adjust the ratio of functional to non-functional transcripts. Shifting between the production of functional and PTC-containing transcripts could be achieved by post-translational modifications of proteins from the splicing machinery such as serine/arginine (SR)-rich splicing factors to facilitate or repress interaction of the spliceosome with a subset of mRNAs.

Another possible mechanism relies on the presence of information in the differentially retained introns or in the flanking exons, such as *cis*-acting intronic splicing silencers that are differentially recognized by *trans*-acting mRNA-binding proteins. In this scenario, post-translational modifications of mRNA-binding proteins could also modulate the probability of splicing events near a given site. In any case, the production of non-functional mRNA isoforms contributes massively to the apparent transcriptome/proteome discordance in plants, but to a much lesser extent in animals where the vast majority of alternative splicing events yield functional products. Quantitative distinction between functional and non-functional transcripts, presently still a challenging task for genome-wide transcriptional surveys, would most probably change the correlation between transcript and protein levels toward a higher concordance correlation coefficients in plants.

## Build to order: do plants produce specialized ribosomes?

Translation is mediated by ribosomes, intricate molecular machines composed of ribosomal RNA and ribosomal proteins (r-proteins) that translate the genetic code encrypted in the DNA into proteins. Because the function of ribosomes is highly conserved in both prokaryotes and eukaryotes, r-proteins are traditionally classified as housekeeping. However, eukaryotic ribosomes contain more proteins than their bacterial counterparts and possess diverse r-RNA modifications that are not found in prokaryotic ribosomes, indicating more sophisticated molecular functions of eukaryotic ribosomes.

In contrast to animals, in which r-proteins are mostly encoded by a single gene, plant r-proteins are encoded by paralogous families comprising several members that generate diverse, functional proteins. For example, the 81 r-proteins of *Arabidopsis* are encoded by more than 200 genes, with each r-protein family consisting of 2–7 members. This does not only complicate coordinate expression of equiamounts of r-proteins to secure ribosomal function, but also allows for a nearly infinite number of differently composed ribosomes, the heterogeneity of which can be further increased by numerous post-translational modifications. In humans and Drosophila, defects in r-protein expression have been associated with diseases (Kongsuwan et al., 1985; Uechi et al., 2001), suggesting functions beyond translation. Similarly, in plants several r-protein mutants are affected in cell division and/or cell expansion resulting in deformed leaves (Rosado et al., 2012), indicating specific, extra-ribosomal functions of some r-proteins in developmental processes. It should be noted that in plants accurate detection of protein concentrations of paralogous proteins by mass spectrometry is rendered difficult as these proteins often have identical sequence parts that cannot be distinguished.

## A plant-specific ribosome code?

The large number of r-protein paralogs in plants invites speculation as to whether populations of structurally diverse ribosomes produced during development or in response to environmental signals can capture mRNAs differentially and prioritize the translation of specific subsets of mRNAs. The relative incorporation of r-protein paralogs is altered by growth conditions at the protein level (Hummel et al., 2012), and transcripts encoding r-protein accumulate differentially upon iron and phosphate deficiency (Rodríguez-Celma et al., 2013; Wang et al., 2013), suggesting that the translational machinery is remodeled in response to environmental signals. Heterogeneous ribosomal populations would contribute markedly to discordant changes in transcript and protein profiles. Translatome profiling studies support a regulatory intervention of protein abundance at the translational level (Mustroph et al., 2009; Juntawong et al., 2014). A transcriptomic comparison of steady-state and polysome-bound mRNAs revealed that translational control is independent of mRNA abundance (Liu et al., 2012). The authors of this study concluded that translational control has a greater effect on gene activity than the high steady state mRNA levels. Dynamic changes in r-protein composition would offer a plausible explanation for the observed differential translational efficiency of mRNAs in response to changing environmental conditions.

## Conclusions

Based on the above considerations, we propose that the uncoupling of transcript and protein abundances in plants is driven by additional mechanisms that are not prominent or not present at all in other organisms. This proposal is supported by the predominant forms of alternative splicing in plants (i.e., intron retention and alternative donor or acceptor splice sites) and a highly dynamic ribosome composition that aids in tuning protein profiles to cellular demands during development or stress. Although ribosomal specificity might also exist in mammals (O'Leary et al., 2013), the large number of r-protein genes suggests that heterogeneity of ribosomes in plants is much more pronounced than in other eukaryotes. Also, recruitment of ribosomes to mRNA may have plant-specific dynamics that affect translation efficiency (Lan and Schmidt, 2011). An underappreciated factor is the cell type-specific variation in splicing patterns (Lan et al., 2013), r-protein composition (Whittle and Krochko, 2009), and other, not yet explored processes that may differ among cell types such as mRNA export, and protein stability, which may contribute to the high mRNA/protein discordance in multicellular organisms. Another factor that has not yet been explored at the whole-genome scale is the impact of microRNAs on translation and thus on mRNA/protein abundance correlation in plants. The points brought up here are not only of academic interest. “True” discordance mirrors an underappreciated regulatory layer for determining the final concentrations of proteins. Attempts to engineer the genetics of crop plants to improve plant performance under stress conditions traditionally aim at the control of gene expression. Knowledge regarding post-transcriptional regulation that contributes to transcript/protein discordance may aid in generating stress-resistant germplasm.

### Conflict of interest statement

The authors declare that the research was conducted in the absence of any commercial or financial relationships that could be construed as a potential conflict of interest.
